# Does physical activity level and total energy expenditure relate to food intake, appetite, and body composition in healthy older adults? A cross-sectional study

**DOI:** 10.1007/s00394-024-03571-z

**Published:** 2025-01-24

**Authors:** Dilara Dericioglu, Lisa Methven, Miriam E. Clegg

**Affiliations:** 1 Hugh Sinclair Unit of Human Nutrition, Department of Food and Nutritional Sciences, University of Reading, Whiteknights, Reading, RG6 6DZ UK; 2 Food Research Group, Department of Food and Nutritional Sciences, University of Reading, Whiteknights, Reading, RG6 6DZ UK; 3 Institute of Food, Nutrition and Health, University of Reading, Whiteknights, Reading, RG6 6EU UK; 4 School of Food and Nutritional Sciences, University College Cork, Cork, Ireland

**Keywords:** Appetite, Physical activity, Total energy expenditure, Body composition, Energy intake, Older adults

## Abstract

**Purpose:**

With ageing, older adults (≥ 65 years) may experience decreased appetite, contributing to declines in body weight and muscle mass, potentially affecting physical capabilities. Physical activity (PA) has been suggested as a potential strategy to enhance appetite in older adults, but evidence supporting this is insufficient. This study aimed to investigate the relationship between PA levels, total energy expenditure (TEE), body composition, energy intake (EI) and appetite in older adults.

**Methods:**

One hundred and eight healthy older adults (age 70 ± 4 years; BMI 24.3 ± 2.6 kg/m^2^) were categorised into three groups (low, medium, high) based on accelerometer-measured PA level (AMPA) and TEE from 7-day PA diaries. Body composition was measured using bioelectrical impedance. Energy and nutrient intakes were assessed using 3-day weighed food diaries. Appetite was assessed using the visual analogue scales at 30-min intervals throughout 1 day.

**Results:**

TEE was positively correlated with EI and % muscle mass (p < 0.05), with higher % muscle mass and TEE associated with higher EI. Energy and protein intake were significantly higher in the high TEE group than the low group (p = 0.03, p = 0.01; respectively). No significant differences in energy and macronutrient intake were observed across AMPA groups, and appetite components (hunger, fullness, desire to eat, prospective consumption) did not differ significantly in either the AMPA or TEE groups.

**Conclusions:**

Higher TEE is associated with higher energy and protein intake, with body composition playing a crucial role. These findings highlight the importance of considering PA, TEE, and body composition in interventions aimed at improving EI in older adults.

**Clinical Trail registration**: clinicaltrials.gov as NCT05067036. Registered 2 October 2021, https://classic.clinicaltrials.gov/ct2/show/NCT05067036

## Introduction

Loss of appetite, often referred to as the anorexia of ageing, is a prevalent issue among older adults [1], affecting, approximately 22% of community-dwelling individuals [2]. This condition can be the result of various physiological (e.g. decreased lean body mass, reduced metabolic rate), pathological (e.g. chronic diseases, depression), or social changes (e.g. loneliness, widowhood) that occur with ageing [3, 4]. As people age, body fat typically increases due to decreased physical activity and a diminished resting metabolic rate whereas fat-free body mass, including muscle mass, decreases [5].

Fat-free mass, which includes both muscle mass and high metabolic rate organs such as brain, liver, and kidneys [6], has a higher energy requirement than fat mass [7]. These components can significantly influence basal metabolic rate, thereby affecting overall energy expenditure [7]. Research indicates that a 1–2% decline in fat-free mass associated with ageing [8] is closely linked to a corresponding decrease of approximately 1–2% in resting metabolic rate over each decade in adults [7]. Cross-sectional studies strongly support the notion that fat-free mass, including muscle mass, plays a crucial role in modulating energy intake and hunger [9]. This influence likely occurs indirectly through its effects on energy expenditure [10] and resting metabolic rate [11], and directly through complex signalling mechanisms between muscle mass and brain involved in appetite regulation [12]. However, this relationship is complicated by the fact that increased muscle mass often correlates with higher levels of physical activity [13], which itself can stimulate appetite [14].

The anorexia of ageing can cause many adverse health outcomes, especially weight loss and malnutrition [15]. Malnutrition, which is associated with reduced muscle function [16], is a serious health concern that disproportionately affects older adults, who may struggle to meet their estimated average energy requirement due to decreased appetite [17]. It has been stated that inadequate food intake associated with anorexia causes a decrease in exercise capacity, muscle mass and strength [18], which are major risk factors for developing frailty and sarcopenia in older adults [19, 20].

It is known that a person’s physical capacity improves with increased physical activity [21]. There is clear evidence that regular and appropriate physical activity in older age has an important role in improving muscle strength, increasing physical functioning, and maintaining an independent lifestyle [22, 23]. It is recommended that older adults (≥ 65 years) do at least 150–300 min of moderate physical activity or at least 75–150 min of vigorous physical activity or an equal combination of moderate to vigorous physical activity per week [24]. Additionally, in the UK, older adults are recommended to take part in combined bone and muscle strengthening activities (e.g. carrying heavy bags, gym, yoga) twice a week to keep muscles, bones, and joints strong; and to do balance activities (e.g. dancing, bowls, Tai Chi) twice per week to reduce the risk of frailty and falls [25]. Despite these recommendations, many older adults fall short, with data showing that physical activity levels decline significantly with age [25, 26].

Decreased physical activity is considered one of the potential factors that may be responsible for appetite reductions as people age further [27]. Therefore, organizations like the NHS and charities such as Age UK recommend physical activity for older adults to help increase appetite [28, 29]. Research on whether physical activity increases appetite and energy intake are divided into two broad categories: studies that examine single bouts of acute exercise and that examine chronic exercise training performed for weeks or months [30]. While there are more studies in the literature focusing on appetite responses to acute exercise, fewer studies have explored the differences in appetite regulation between physically active and sedentary individuals. A systematic review that included some of these studies has reported no consistent differences in appetite or absolute energy intake between active and sedentary groups [14]. In the general adult population, the relationship between habitual physical activity and energy intake has been proposed to follow a J-shaped curve, where energy intake is high at very low physical activity (non-regulated), reduces to a minimum at low physical activity and then increases with increasing activity (regulated) [14]. However, authors have suggested that this J-shaped relationship may not hold true for older adults; they have proposed a distorted relationship where energy intake is very low at very low physical activity (presenting as a severely impaired drive to eat), and subsequently energy intake increases with increasing physical activity, but not at a proportional rate [31–33]. This proposed relationship emphasizes the need for further investigation to uncover the optimal levels of physical activity that may promote appetite regulation among older adults. Overall, a systematic review indicated that while frequent physical activity improves appetite control in younger adults [14], its effect on older adults remains less evident [34, 35].

In this context, understanding the impact of total energy expenditure (TEE) on appetite and energy intake is essential, as it is influenced by physical activity and resting metabolic rate [36]. While emphasizing the importance of physical activity for older adults, the relationships between TEE and energy intake in this population has had limited exploration [37]; particularly concerning their specific effect on appetite. Therefore, the overall aim of this study is to investigate the relationships between physical activity, TEE, body composition, energy intake, appetite, and eating behaviour in older adults (≥ 65 years).

## Methods

### Study design and participant criteria

The study had a cross-sectional study design and was undertaken over 7 continuous days. The research protocol was approved by the University of Reading Research Ethics Committee (UREC No 20/32); Clinical Trials Database Registration ID NCT05067036) and the study was conducted in participant’s homes due to COVID-19 restrictions.

One hundred and eight older adults (≥ 65 years) from Reading, Surrey, and surrounding areas participated in the study between December 2020 and May 2021. The inclusion criteria were as follows: healthy (free from chronic diseases such as cardiovascular disease, diabetes, thyroid disorders, cancer, heart, lung, and kidney disease); not living with obesity (Body mass index (BMI) < 30 kg/m^2^); not using a medication that can impact on appetite, food intake or body weight in the past 3 months; not changing their diet, exercise or physical activity level, and not having unexpected weight loss in the last 3 months; living independently; being able to comprehend the study procedures; and not smoking more than ten cigarettes a day.

#### Pre-screening

Prior to participation, participants were sent an information sheet and were asked to complete a health and lifestyle questionnaire to determine their eligibility. Those who met the inclusion criteria received a phone call during which the study details were explained in full, after which informed consent was obtained both verbally and via e-mail.

#### Test days

Following this, participants were posted or delivered a study box containing a series of self-administered questionnaires, an accelerometer (AX3, 3-Axis Logging Accelerometer; Newcastle, United Kingdom), a tape measure, a bioelectrical impedance scale (OMRON VIVA Smart Scale and Body Composition Monitor—HBF-2222T-EBK; United Kingdom), a simplified physical activity record paper (sPAR) [38], printed food diaries, and appetite rating scales (paper booklet) [100 mm visual analogue scale (VAS)]. A digital kitchen scale was also provided to those who did not have one at home. Prior to delivery, participants were requested to watch the video instructions on how to use the equipment and complete the questionnaires and papers. An instruction sheet was also included in the study box. Further assistance was also provided via email, phone call, or video chat whenever required. After 7 days, one of the researchers collected the study box from the participant’s home.

### Outcome variables

#### Assessment of eating behaviour, appetite, physical activity, nutritional knowledge, and frailty

Participants were asked to complete questionnaires pertaining to appetite, physical activity, eating behaviour, nutritional knowledge and frailty; these were the Council on Nutrition Appetite Questionnaire (CNAQ) [39], the Dutch Eating Questionnaire (DEBQ) [40], the Three Factor Eating Questionnaire (TFEQ) [41], the Control of Eating Questionnaire (CoEQ) [42], the General Practice Physical Activity Questionnaire (GPPAQ) [43], as well as the Physical Activity Scale for the Elderly (PASE) [44], a nutritional knowledge questionnaire (General Nutrition Knowledge Questionnaire (GNKQ) [45]), and a frailty indicator (Groningen Frailty Indicator Questionnaire (GFI) [46].

#### Measurement of body composition and physical activity level

Participants were asked to complete body composition measurements on one occasion during the study period, after waking up and while fasted (before having breakfast and consuming water). They were asked to measure their height, waist, and hip circumference (in cm) using the tape measure and weigh themselves using the bioelectrical impedance scale for measurements of body weight (in kg), percentage body fat and muscle mass, and visceral fat. They recorded the results on the provided body composition record sheet.

Participants’ physical activity levels were measured using accelerometers. They were instructed to wear the accelerometer in the elastic waterproof wristband provided on their non-dominant wrist 24 h a day for seven consecutive days [47]. The accelerometers were set up using the OMGUI software to record raw, triaxial acceleration at a rate of 100 Hz and a dynamic range of ± 8 g, measuring min per day spent in activities of four different intensities: sedentary (< 1.5 METS), light (≥ 1.5 METS, < 4 METS), moderate (≥ 4 METS, < 7 METS), and vigorous (≥ 7 METS) [48]. The raw triaxial data were summarised into a signal magnitude vector (gravity-subtracted) (SVMgs) using 1-s epochs. The signal was filtered for only frequencies of human movement, by applying a fourth-order Butterworth band-pass filter between 0.5 and 20 Hz. Cut-offs were applied based on Esliger et al., these were 217 g min for light activity, 645 g min for moderate activity and 1811 g min for vigorous activity [49]. Participants’ time spent in moderate and vigorous intensity activities (min/day) was calculated as tertiles within each sex, and then combined into overall low, medium, and high groups.

Additionally, to calculate TEE, participants were asked to complete a sPAR while wearing the accelerometers. The sPAR, previously validated for use in the adult population [50] and modified to fit the older adult population [38] included categories for transportation, daily life activities, leisure activities, and sports activities. Participants were asked to fill out this paper in 15-min intervals across the 7 days. If the activity type was not listed on the sheet, they were instructed to describe the activity, along with its duration and type, in the bottom section. These recordings were then analysed using an Excel spreadsheet template created by Gerrior et al. [51], which uses the Estimated Energy Requirement equations of the Dietary Reference Intake Committee. The template includes data entry points for the individual’s age (in years), weight (in kilograms), height (in meters), sex, a list of activities performed and their intensity (Metabolic Equivalent of Task; MET), and the duration (min) of each activity in the past 24 h. TEEs are calculated automatically based on these data. We modified the activity list in this template according to the sPAR and updated the MET values with the current ones [52]. Participants were then divided into tertiles within each sex based on the TEE data and combined into low, medium, and high groups.

#### Assessment of food intake and appetite

Participants were instructed in methods to accurately record their food and beverage intake for 3 days (2 weekdays and 1 weekend day) using a 24-h weighed food diary while wearing the accelerometer. Each food diary was analysed by the same researcher using the Nutritics software (Nutrition Analysis Software for Professionals; Dublin, Ireland) to estimate energy, macronutrient, and fibre intakes.

Additionally, participants were asked to complete the appetite rating scales hourly during waking hours for 1 day, while wearing the accelerometer and keeping a food diary record. Four subjective feelings (hunger, fullness, desire to eat, and prospective consumption) of appetite was measured using a 100 mm VAS, anchored with the terms ‘not at all’ and ‘extremely’ [53]. The change from baseline (the first-fasted value of the day) in VAS score of appetite was calculated and, subsequently, the area under the curve (AUC) for each variable was calculated using the trapezoidal rule.

### Statistical analysis

In total, 108 older participants were recruited into the study. This study size was based on a cross-sectional study examining the effect of habitual physical activity on energy compensation [54]. Based on a significant difference in energy intake of 486.8 kcal between groups and a standard deviation of 612.85 kcal, including a power of 0.9 and a α = 0.05, a total of 108 participants were required consisting of 3 groups of 36 (low, medium, and high).

Statistical analysis was performed using SPSS (version 27; Chicago, Illinois, United States). All data were first tested for normal distribution using the Shapiro–Wilk test and all values were expressed as means ± standard deviation. P-values < 0.05 were considered significant in all analyses. A Two-way Analysis of Covariance (ANCOVA) test was performed to compare the participants’ characteristics, questionnaire scores, intakes (energy, macronutrient, and fibre), and the VAS scores for appetite between the accelerometer measured physical activity (AMPA) (high, medium, low) and TEE groups (high, medium, low) assessed using sPAR, using sex as a fixed factor and age as a covariate. Additionally, baseline VAS scores were included as covariates in the analysis for appetite. Where a significant difference was found, pairwise comparisons with a Bonferroni correction were completed. Where data was not normally distributed, a log transformation was applied before conducting the ANCOVA.

Pearson’s (for parametric data) or Spearman’s (for non-parametric data) correlation coefficients (r and r_s_ respectively), as appropriate, were used to assess the association of the participants’ body composition (% muscle and fat mass) and TEE with intakes (energy, macronutrients, and fibre), and appetite. Subsequently, a hierarchical multiple linear regression analysis was conducted to investigate the independent associations of % muscle mass, % fat mass, and physical activity level with TEE, controlling for sex and age. This analysis was also performed to examine the independent associations of % muscle mass, % fat mass, physical activity level, and TEE with energy intake, while adjusting for sex and age. Age and sex were entered into the model as predictors in the first step, followed by % muscle mass, % fat mass, physical activity level, and TEE in the second step to assess their independent contributions. Multicollinearity was tested using Variance Inflation Factor (VIF) scores, which were all between 1 and 5, indicating acceptable levels of correlation between the predictor variables, and ensuring the reliability of the model.

## Results

### Participants’ characteristics

**Fig. 1:**
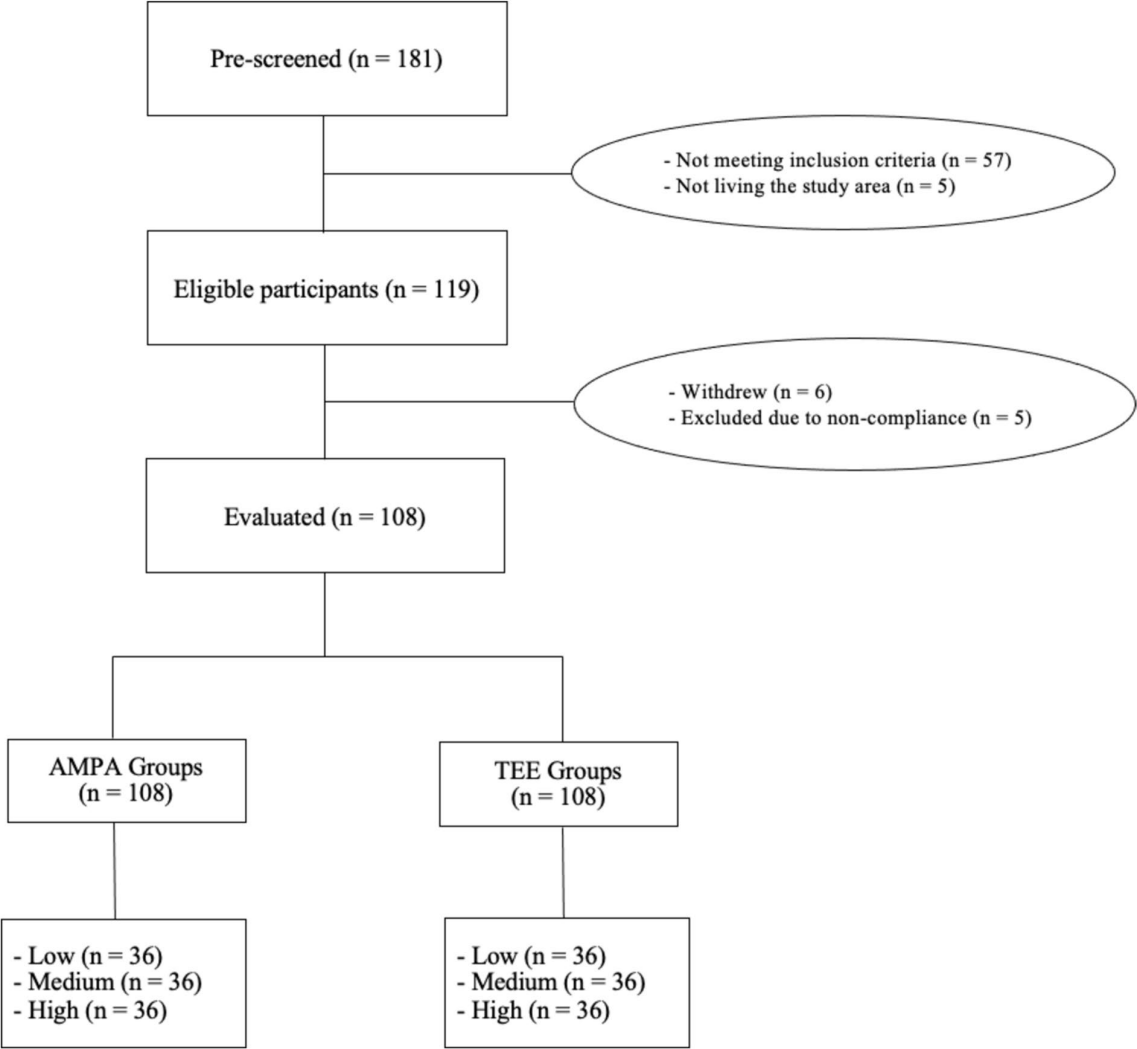
A flow diagram of the participant recruitment

Of 181 pre-screened participants, 57 did not meet the study inclusion criteria and 5 were not living in the study area (Reading, Surrey, and surrounding areas), so study equipment could not be delivered or posted to them. Another 6 withdrew before completing the study, and 5 were excluded due to non-compliance (Fig. 1).

After completion of data collection, as mentioned above, participants were divided into three groups as low, medium, and high according to AMPA (< 100.86 min/day; 102.71–127.86 min/day, > 128 min/day, respectively) and TEE (< 2268.8 kcal/day; 2274.1–2434.9 kcal/day; > 2438.4 kcal/day, respectively) assessed by sPAR (Fig. 1). For two participants there were issues with the accelerometer and only 3 days data was recorded. For all other participants the average of the 7 days data was used. The participants’ characteristics are shown in Table 1**.** There were no significant differences in weight, BMI, waist and hip circumferences, % fat and muscle mass, or visceral fat across the AMPA groups (p > 0.05). While age was significantly higher in the low AMPA group than the high AMPA group (p = 0.01), no significant difference was observed among the TEE groups (p > 0.05). Height, weight, BMI, hip and waist circumferences, % fat mass, and visceral fat were significantly higher in the high TEE group compared to the low and medium TEE groups (p < 0.05), while % muscle mass was significantly higher in the low and medium TEE groups compared to the high TEE group (p < 0.05). Additionally, height and weight in the low TEE were significantly lower than those in the medium TEE group (p < 0.05). Furthermore, there was no significant interaction effect between the AMPA and TEE groups and sex on any of the variables (e.g., height, weight, BMI) (p > 0.05), indicating that the effects of AMPA and TEE groups on these variables did not differ by sex.

**Table 1:** Participants’ characteristics

	Overall (*n* = 108)	LoAMPA (*n* = 36)	MeAMPA (*n* = 36)	HiAMPA (*n* = 36)	Sig *p*-value^*^	LoTEE (*n* = 36)	MeTEE (*n* = 36)	HiTEE (*n* = 36)	Sig *p*-value^*^
Age (years)	70 ± 4	71 ± 4^a^	70 ± 4	68 ± 3	0.01	71 ± 5	70 ± 4	69 ± 3	0.26
Male/female (n)	49/59	16/20	16/20	17/19	0.96	16/20	16/20	17/19	0.96
Height (cm)	168.6 ± 10.0	169.4 ± 8.9	169.0 ± 10.5	167.3 ± 10.7	0.14	163.5 ± 8.9^de^	168.8 ± 8.7^d^	173.4 ± 10.0	< .001
Weight (kg)	69.5 ± 12.4	71.0 ± 11.8	68.4 ± 12.3	69.2 ± 13.1	0.59	62.5 ± 10.9^df^	66.7 ± 8.1^d^	79.4 ± 11.2	< .001
BMI (kg/m^2^)	24.3 ± 2.6	24.7 ± 2.5	23.7 ± 2.5	24.5 ± 2.6	0.26	23.2 ± 2.6^d^	23.4 ± 1.9^d^	26.3 ± 2.0	< .001
WC (cm)	87.8 ± 9.9	89.9 ± 10.1	86.4 ± 8.7	87.0 ± 10.7	0.23	83.8 ± 9.8^d^	86.0 ± 7.7^d^	93.5 ± 9.6	< .001
HC (cm)	99.0 ± 5.8	101.1 ± 5.5	98.0 ± 5.9	97.7 ± 5.6	0.06	96.3 ± 6.0^d^	97.3 ± 3.6^d^	103.3 ± 5.0	< .001
Fat (%)	28.5 ± 8.0	29.7 ± 8.0	27.4 ± 8.2	28.4 ± 7.8	0.30	26.2 ± 8.4^d^	27.7 ± 7.1^ g^	31.6 ± 7.5	< .001
Muscle (%)	27.0 ± 4.9	26.3 ± 4.7	27.4 ± 4.9	27.4 ± 4.9	0.18	27.4 ± 5.5^ g^	27.6 ± 4.6^ g^	26.1 ± 4.2	0.01
Visceral Fat	8.1 ± 2.8	8.6 ± 3.0	7.8 ± 2.8	8.1 ± 2.7	0.51	7.3 ± 2.8^d^	7.1 ± 1.9^d^	10.0 ± 2.8	< .001
AMPA (min/day)	124 ± 50	78 ± 19^bc^	118 ± 11^b^	177 ± 43	< .001	121 ± 50	120 ± 44	132 ± 54	0.63
TEE (kcal/day)	2732 ± 486	2734 ± 446	2683 ± 501	2778 ± 515	0.31	2427 ± 328^de^	2732 ± 420^d^	3036 ± 496	< .001

*BMI* Body mass index; *WC* Waist circumference; *HC* Hip circumference; *AMPA* Accelerometer measured physical activity level; *TEE* Total energy expenditure

Physical Activity groups: Low (LoAMPA), Medium (MeAMPA), High (HiAMPA)—measured by accelerometer

TEE groups: Low (LoTEE), Medium (MeTEE), High (HiTEE)—assessed using sPAR

^*^Data were analysed by Two-Way ANCOVA (controlling for sex as a fixed factor and age as a covariate) except sex which was analysed with chi-square test. Values are means ± SD

^a^ p < 0.05 compared to HiAMPA, ^b^p < 0.001 compared to HiAMPA, ^c^p < 0.001 compared to MeAMPA

^d^p < 0.001 compared to HiTEE, ^e^p < 0.001 compared to MeTEE, ^f^p < 0.05 compared to MeTEE, ^g^p < 0.05 compared to HiTEE

### Differences in appetite, eating behaviour, nutritional knowledge, and frailty between AMPA and TEE groups

The appetite level of the participants was assessed using the CNAQ, with a score below 28 indicating poor appetite. On average, none of the groups exhibited poor appetite, and no significant differences in appetite scores were found between the groups (p > 0.05). Restrained eating behaviour was measured using the DEBQ and the TFEQ, with a score over 2.5 on the DEBQ and a score over 10 on the TFEQ indicating restrained eating. There were no significant differences in restrained eating behaviour between the groups according to either the TFEQ or DEBQ (p > 0.05). Additionally, no significant differences were found between the groups in terms of general or specific food cravings, as measured by the CoEQ (p > 0.05). While there were no significant differences in AMPA levels between the groups based on the GPPAQ and PASE questionnaire (p > 0.05), TEE was positively correlated with PASE scores (r_s_(106) = 0.22, p = 0.024). In contrast, no significant correlations were observed between AMPA levels and either TEE or PASE scores (p > 0.05). Furthermore, no significant differences were found between the groups for frailty levels assessed using the GFI, or for nutritional knowledge as determined by the GNKQ (p > 0.05) (Table 2).

**Table 2:** Differences in appetite, eating behaviour, nutritional knowledge, and frailty between AMPA and TEE groups

	Overall (*n* = 108)	LoAMPA (*n* = 36)	MeAMPA (*n* = 36)	HiAMPA (*n* = 36)	Sig *p*-value^*^	LoTEE (*n* = 36)	MeTEE (*n* = 36)	HiTEE (*n* = 36)	Sig *p*-value^*^
CNAQ	31.0 ± 2.1	30.7 ± 1.9	31.1 ± 2.4	31.0 ± 2.2	0.75	30.7 ± 2.1	30.8 ± 2.6	31.4 ± 1.7	0.51
Score (17–28) (%)	9	11	14	3		11	14	3	
DEBQ	2.5 ± 0.8	2.6 ± 0.7	2.3 ± 0.8	2.7 ± 0.7	0.11	2.3 ± 0.6	2.6 ± 0.9	2.7 ± 0.7	0.06
Restraint (score > 2.5) (%)	52	58	39	58		33	58	64	
TFEQ	7.7 ± 4.3	7.8 ± 4.5	7.1 ± 4.3	8.2 ± 4.3	0.60	6.8 ± 4.2	8.3 ± 4.4	8.0 ± 4.4	0.40
Restraint (score > 10) (%)	30	25	28	36		19	42	28	
CoEQ (General)	14.2 ± 8.3	11.7 ± 8.1	15.8 ± 8.3	15.2 ± 8.2	0.18	14.5 ± 8.9	15.1 ± 8.2	13.1 ± 8.0	0.46
CoEQ (sweet)	7.5 ± 4.4	7.1 ± 4.4	7.8 ± 3.9	7.7 ± 4.8	0.86	7.6 ± 5.0	7.9 ± 4.5	7.1 ± 3.5	0.66
CoEQ (savoury)	2.9 ± 2.5	2.6 ± 2.6	2.9 ± 2.5	3.2 ± 2.5	0.61	3.2 ± 3.0	2.2 ± 2.1	3.3 ± 2.3	0.15
CoEQ (dairy)	3.8 ± 2.5	3.8 ± 2.4	3.8 ± 2.6	3.9 ± 2.6	0.97	3.3 ± 2.3	4.0 ± 2.8	4.2 ± 2.4	0.39
GNKQ	70.7 ± 8.4	69.0 ± 9.5	71.1 ± 8.5	72.1 ± 6.9	0.51	70.0 ± 8.9	70.8 ± 9.1	71.4 ± 7.4	0.88
GFI	1.6 ± 1.6	1.7 ± 1.5	1.7 ± 1.7	1.5 ± 1.6	0.92	1.7 ± 1.7	1.6 ± 1.5	1.6 ± 1.5	0.95
GPPAQ^!^					0.55				0.35
Inactive (%)	48	53	44	47		56	47	42	
Moderately Inactive (%)	6	3	8	6		0	11	6	
Moderately Active (%)	24	30	17	25		28	17	27	
Active (%)	22	14	31	22		16	25	25	
PASE^!^	149 ± 59	143 ± 63	147 ± 54	158 ± 62	0.58	143 ± 58	167 ± 67	138 ± 50	0.09
Sedentary (score 0–40) (%)	1	3	0	0		3	0	0	
Light physical activity (score 41–90) (%)	14	19	11	11		14	11	17	
Moderate to intense activity (score > 90) (%)	85	78	89	89		83	89	83	

*CNAQ* Council on Nutrition Appetite Questionnaire; *DEBQ* Dutch Eating Behaviour Questionnaire; *TFEQ* Three-Factor Eating Questionnaire; *CoEQ* Control of Eating Questionnaire; *GNKQ* General Nutrition Knowledge Questionnaire; *GFI* Groningen Frailty Indicator Questionnaire; *GPPAQ* General Practice Physical Activity Questionnaire; *PASE* Physical Activity Scale for the Elderly; *BMI* Body mass index

Physical Activity groups: Low (LoAMPA), Medium (MeAMPA), High (HiAMPA)—measured by accelerometer

Total Energy Expenditure (TEE) groups: Low (LoTEE), Medium (MeTEE), High (HiTEE)—assessed using sPAR

^**^Data were analysed by Two-Way ANCOVA (controlling for sex as a fixed factor and age as a covariate). ^!^Data were analysed by Chi-square test. Values are means ± SD

### Differences in energy and nutrients intakes between AMPA and TEE groups

No significant difference was observed in energy, protein, carbohydrate, and fat intake between the AMPA groups (p > 0.05), whereas a significant difference was found in fibre intake (F (2,101) = 3.105, p = 0.049). Pairwise comparisons indicated a trend towards higher fibre intake in the high AMPA group compared to the low AMPA group, though this difference did not reach statistical significance (p = 0.053). For the AMPA groups, sex had a significant main effect on energy (F (1,101) = 49.156, p < 0.001), carbohydrate (F (1,101) = 31.436, p < 0.001), protein (F (1,101) = 39.096, p < 0.001), fat (F (1,101) = 22.864, p < 0.001), and fibre intake (F (1,101) = 7.827, p = 0.01), with males consistently showing higher intake levels compared to females across all nutrients (p < 0.001). Furthermore, an interaction effect between AMPA groups and sex was also observed for protein intake (F (2,101) = 6.654, p = 0.002), indicating that the difference in protein intake between AMPA groups varied by sex. Specifically, males in the high and medium AMPA groups had significantly higher protein intake compared to females in the same groups (p < 0.001, p = 0.002, respectively). Additionally, males in the high AMPA group had significantly higher protein intake than those in the low and medium AMPA groups (p < 0.001, p = 0.03, respectively).

For the TEE groups, significant main effects were observed for energy intake (F (2,101) = 3.642, p = 0.03) and protein intake (F (2,101) = 5.246, p = 0.007), showing that energy and protein intake in the high TEE group were significantly higher than in the low TEE group (p = 0.025, p = 0.006, respectively). Additionally, sex had a significant main effect on intakes of energy (F (1,101) = 52.055, p < 0.001), carbohydrate (F (1,101) = 32.362, p < 0.001), protein (F (1,101) = 37.420, p < 0.001), fat (F (1,101) = 23.667, p < 0.001), and fibre (F (1,101) = 7.470, p = 0.007) in the TEE groups, with males showing higher intake levels across all nutrients compared to females. An interaction effect between TEE groups and sex was observed for energy (F(2, 101) = 4.527, p = 0.013) and carbohydrate intake (F(2, 101) = 4.244, p = 0.017), indicating that the differences in energy and carbohydrate intake between TEE groups varied by sex. Specifically, males in the high TEE group had significantly higher energy and carbohydrate intake compared to those in the medium and low TEE groups (p < 0.05). Additionally, males across all TEE groups had significantly higher energy and carbohydrate intakes compared to females in the same TEE groups (p < 0.05) (Table 3).

**Table 3:** Differences in energy, macronutrient, and fibre intake between AMPA and TEE groups

	Overall (*n* = 108)	LoAMPA (*n* = 36)	MeAMPA (*n* = 36)	HiAMPA (*n* = 36)	Sig *p*-value^*^	LoTEE (*n* = 36)	MeTEE (*n* = 36)	HiTEE (*n* = 36)	Sig *p*-value^*^
Energy (kcal)	1883 ± 421	1802 ± 332	1874 ± 409	1973 ± 498	0.129	1774 ± 308^b^	1881 ± 421	1994 ± 494	0.030
Carbohydrate (g)	210 ± 54	198 ± 47	209 ± 51	222 ± 61	0.087	200 ± 47	205 ± 49	224 ± 62	0.072
Protein (g)	76 ± 19	73 ± 11	75 ± 18	81 ± 23	0.092	70 ± 14^c^	78 ± 19	82 ± 20	0.007
Fat (g)	75 ± 22	74 ± 17	75 ± 22	77 ± 25	0.855	71 ± 18	75 ± 23	79 ± 23	0.286
Fibre (g)	25 ± 8	23 ± 7^a^	24 ± 8	27 ± 9	0.049	26 ± 8	25 ± 8	25 ± 8	0.466

Physical Activity groups: Low (LoAMPA), Medium (MeAMPA), High (HiAMPA)—measured by accelerometer

Total Energy Expenditure (TEE) groups: Low (LoTEE), Medium (MeTEE), High (HiTEE)—assessed using sPAR

^**^Data were analysed by Two-Way ANCOVA (controlling for sex as a fixed factor and age as a covariate). Values are means ± SD

^a^p = 0.053 compared to HiAMPA, ^b^p = 0.025 compared to HiTEE, ^c^p = 0.006 compared to HiTEE

### Difference in appetite scores (VAS) between AMPA and TEE groups

**Fig. 2:**
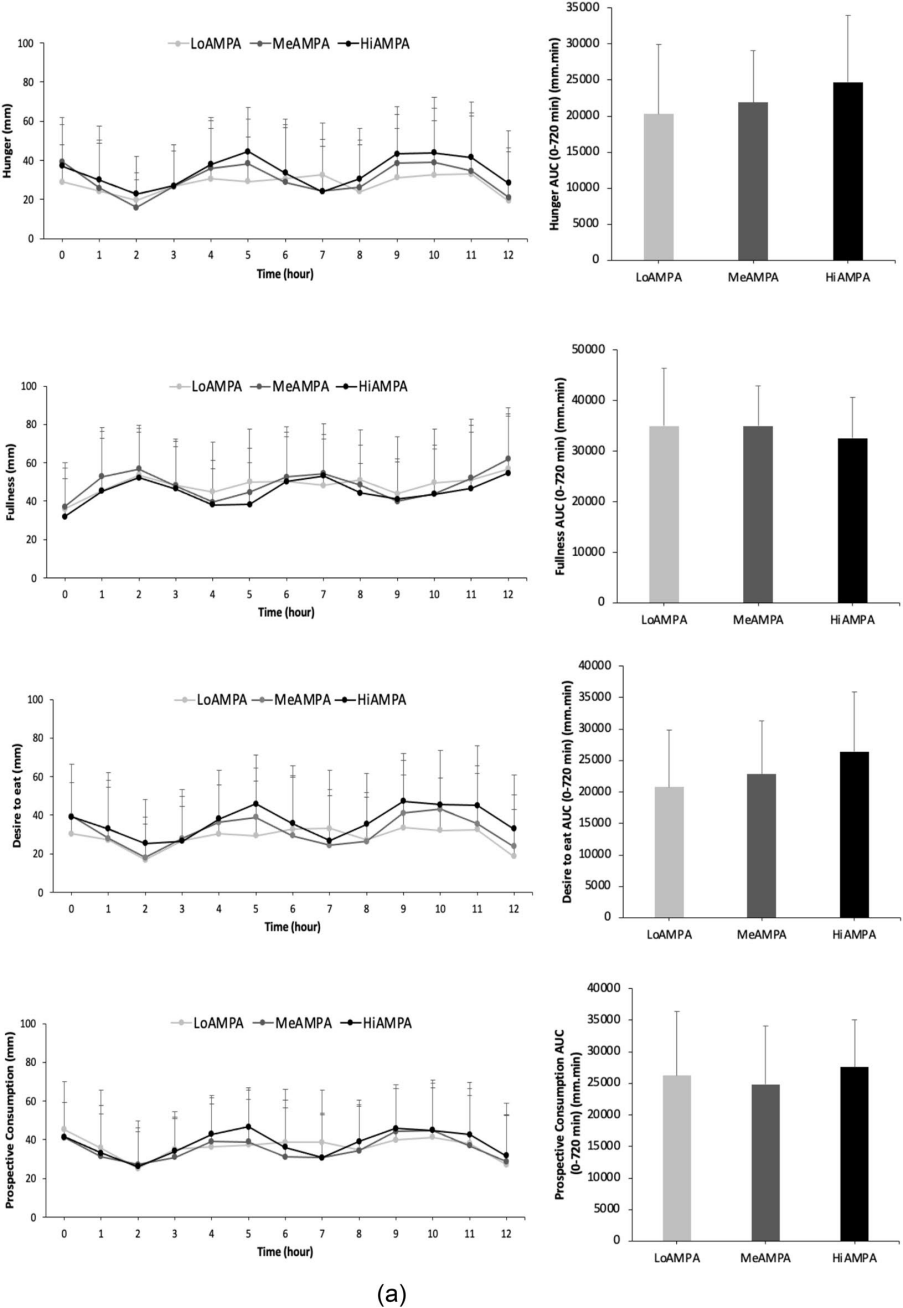

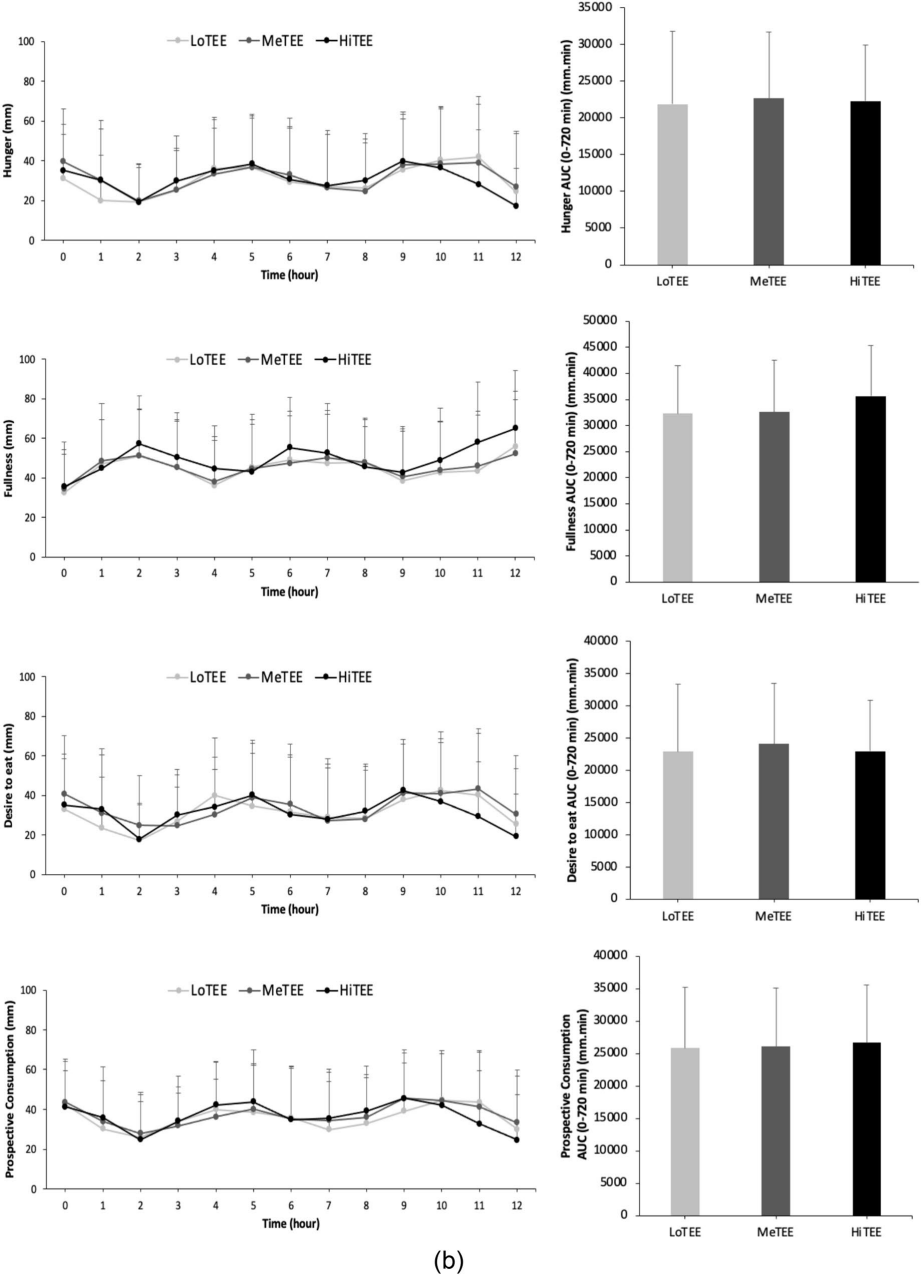
**a** The VAS score of hunger, fullness, desire to eat, and prospective consumption for 1 day while participants were wearing the accelerometer and were keeping the food diary record, as well as the AUC values of VAS scores during the day (0–720 min) for high (HiAMPA), medium (MeAMPA), and low physically active group (LoAMPA). Values are means, with standard deviation represented by vertical bars. **b** The VAS score of hunger, fullness, desire to eat, and prospective consumption for 1 day while participants were filling out the sPAR and were keeping the food diary record, as well as the AUC values of VAS scores during the day (0–720 min) for high (HiTEE), medium (MeTEE), and low activity energy expenditure group (LoTEE). Values are means, with standard deviation represented by vertical bars

There were no significant differences in the total AUC values (0–720 min) of hunger, fullness, desire to eat food, or prospective consumption across the AMPA groups. However, for the AMPA groups, sex had a significant main effect on desire to eat (F (1,97) = 4.698, p = 0.033), indicating that females had a higher desire to eat compared to males. Similarly, there were no significant differences in the total AUC values (0–720 min) of hunger, fullness, desire to eat food, or prospective consumption across the TEE groups. For the TEE groups, sex also had a significant main effect on desire to eat (F (1,97) = 4.612, p = 0.034), with females showing higher desire to eat scores compared to males (Fig. 2a, b).

### Relationships between body composition (percentage muscle and fat mass), AMPA, and TEE with energy, nutrient intake, and appetite: A hierarchical multiple regression analysis

There was a significant positive correlation between TEE and % muscle mass (r_s_(106) = 0.56, p < 0.001), and a significant negative correlation between TEE and % fat mass (r(106) = −0.38, p < 0.001). There was no significant correlation between TEE and AMPA (p > 0.05). Additionally, a hierarchical regression analysis was conducted to investigate if TEE can be predicted by % muscle mass, % fat mass, and AMPA. The overall regression model predicted approximately 68% of the variance in TEE (R^2^ = 0.68, F(5,99) = 42.18, p < 0.001). Age and sex predicted approximately 57% of the variance in TEE, with sex being the only significant predictor, showing higher TEE in males. After controlling for age and sex, Step 2 predicted an additional 11% of the variance in TEE. In this step, only percentage fat mass significantly predicted TEE, with higher percentage fat mass being associated with higher TEE, despite the initial negative correlation (Table 4).

**Table 4:** Regression analysis showing percentage muscle mass, percentage fat mass, and AMPA as predictors of TEE

	Cumulative		Simultaneous	
	R^2^ change	*F*-change	β	p
**TEE** Step 1 Age Sex	0.57	F(2,102) = 68.83^*^	− 0.069 − 1.03	0.258 < 0.001
Step 2 % muscle mass % fat mass AMPA	0.11	F(3,99) = 10.97^*^	0.131 0.535 0.674	0.331 < 0.001 0.502

AMPA; Accelerometer measured physical activity; TEE Total energy expenditure *p < 0.001

There was a significant positive correlation between percentage muscle mass and energy, carbohydrate, protein, fat, and fibre intake (p < 0.001), whereas percentage fat mass was negatively correlated with energy, carbohydrate, protein, fat, and fibre intake (p < 0.001) (Table 5). Additionally, TEE was significantly positively correlated with energy, carbohydrate, protein, fat (p < 0.001), and fibre intake (p = 0.033). Although AMPA did not correlate significantly with energy, protein, or fat intake (p > 0 0.05), it was positively correlated with carbohydrate (p = 0.013) and fibre intake (p = 0.002). Furthermore, there were no significant correlations between percentage muscle mass, fat mass, AMPA or TEE and the total AUC values of hunger, fullness, desire to eat food, or prospective consumption (p > 0.05) (Table 5).

**Table 5:** Correlation of body composition measurements, TEE, and AMPA with energy, macronutrient, fibre intake, and appetite

	% Fat mass	% Muscle mass	TEE (kcal)	AMPA (min/day)
Energy (kcal)	− 0.435**	0.603**	0.530**	0.169
CHO (g)	− 0.336**	0.483**	0.433**	0.239*
Protein (g)	− 0.321**	0.509**	0.534**	0.162
Fat (g)	− 0.384**	0.481**	0.380**	0.048
Fibre (g)	− 0.369**	0.400**	0.205*	0.295**
Total AUC for hunger	− 0.017	0.016	− 0.089	0.136
Total AUC for fullness	0.047	0.059	0.079	− 0.056
Total AUC for desire to eat food	0.043	− 0.035	− 0.116	0.176
Total AUC for prospective consumption	− 0.116	0.169	0.078	0.037

*AMPA* Accelerometer measured physical activity, *TEE* Total energy expenditure, *CHO* Carbohydrate, *AUC* Area under the curve. Data were analysed by Pearson’s correlation excluding percentage muscle mass, AMPA, and TEE analysed with Spearman’s correlation. * p < 0.05, ** p < 0.001

Additionally, a hierarchical regression analysis was conducted to examine the effects of % muscle mass, % fat mass, AMPA, and TEE on energy intake. In the first step of the regression, age and sex were entered as predictors. In the second step, % muscle mass, % fat mass, AMPA, and TEE were added to the model. The overall regression model predicted approximately 40% of the variance in energy intake (R^2^ = 0.40, F(6,98) = 10.82, p < 0.001). Age and sex predicted approximately 32% of the variance in energy intake, but these variables were not significant. After controlling for age and sex, Step 2 predicted an additional 8% of the variance in energy intake. In this step, TEE was a significant predictor of energy intake, with higher % TEE scores associated with higher energy intake (Table 6).

**Table 6:** Regression analysis showing body composition (percentage muscle mass and percentage fat mass), TEE and AMPA as predictors of energy intake

	Cumulative		Simultaneous	
	R^2^ change	*F*-change	β	p
**Energy** Step 1 Age Sex	0.32	F(2,102) = 23.41^*^	0.031 − 0.073	0.717 0.701
Step 2 % muscle mass % fat mass TEE AMPA	0.08	F(4,98) = 3.42^*^	0.355 0.030 0.292 0.147	0.058 0.864 0.038 0.076

*AMPA* Accelerometer measured physical activity, *TEE* Total energy expenditure. *p < 0.001

## Discussion

This study aimed to examine the relationships between older adults’ physical activity levels, TEE, energy intake, appetite, and body composition. % Muscle mass had a positive relationship with TEE, but after controlling for age and sex, fat mass emerged as the stronger predictor of TEE, despite its initial negative correlation. TEE was a significant predictor of energy intake, with higher TEE being associated with greater energy intake. Energy and protein intake were significantly higher in the high TEE group compared to the low TEE group. Appetite did not differ significantly within the AMPA or TEE groups, as assessed by both the CNAQ and VAS scores; however, females had a higher desire to eat compared to males in both groups.

The benefits of moderate to vigorous activity for older adults' health are well documented [21, 55]. However, older adults often engage in lighter-intensity activities such as light walking or housework [56], and these activities may be considerably more strenuous for many sedentary older individuals than younger, fit individuals [55, 57]. Therefore, the intensity of older adults’ habitual physical activity may be underestimated by the standard defined cut-off points for accelerometer-based moderate-intensity activity [58]. Thus, in the present study, physical activity diaries were also used to examine in more detail how long different types of activity were performed, in addition to accelerometer data. The data obtained from here were used to calculate TEE. Accordingly, to examine this topic from a wider perspective and to shed light on the contradictory results in the literature, groups were made not only on physical activity levels (AMPA), but also on TEE. Additionally, the PASE questionnaire, which is specific to healthy older adults, was used to provide additional context for participants’ physical activity level. While AMPA and TEE were not significantly correlated, PASE scores showed a significant correlation with TEE; however, no differences in PASE scores were found between the groups. This aligns with the understanding that accelerometers primarily capture movement and may not effectively record certain activities, such as weight-bearing or arm movements. Consequently, discrepancies between TEE assessed with sPAR, physical activity levels measured by accelerometers, and self-reported PASE scores are expected and have been widely reported in the literature [49, 59–61]. Interestingly, TEE values were relatively similar across AMPA groups, despite significant differences in moderate-to-vigorous physical activity minutes. This likely reflects differences in methodology and individual variability. Additionally, differences in weight and height, integral components of TEE calculation, may have contributed to these results. These findings highlight a critical limitation of relying solely on a single method to assess physical activity in older adults. Self-reported tools like PASE, sPAR may capture activities overlooked by accelerometers, while accelerometer data offer objective insights into movement patterns. Together, these methods underscore the complexity of accurately measuring physical activity in older adult population.

Ageing is a major factor contributing to the decline in muscle mass, and many modifiable factors, such as physical activity and nutrition, can affect the severity and speed of this decline [62]. Despite the known benefits of physical activity in helping older adults maintain a healthy weight, muscle strength, and physical functioning [55, 63], the relationships between different physical activity intensities, physical function, and body composition in this group remain unclear [64]. A study conducted with both younger and older individuals demonstrated that active subjects had lower BMI and body fat percentage compared to inactive subjects [65]. Supporting this, a recent study focusing on individuals aged 70–85 years, which measured body composition using Dual-energy X-ray absorptiometry (DEXA), showed a positive correlation between lower fat percentage and physical activity of any intensity [64]. Similar results have also been shown in studies examining the relationship between energy expenditure and muscle mass, indicating that older adults with higher energy expenditure are more likely to have more lean body mass [66]. However, in the present study, although % muscle mass showed a positive correlation with TEE, higher % fat mass emerged as the stronger predictor of higher TEE after controlling for age and sex, despite its initial negative correlation. This unexpected finding likely reflects the complex interplay between body composition, age and sex in influencing energy expenditure. The inclusion of age and sex as covariates in the regression analysis accounted for their confounding effects, particularly as sex was a strong predictor of TEE, with males exhibiting higher TEE than females. After accounting for these factors, % fat mass contributed more significantly to higher TEE, possibly due to its association with total body mass. Carrying a larger body mass requires more energy, which may explain why individuals with higher % fat mass tend to have higher total energy expenditure [67], even if their activity is similar to that of leaner counterparts [68]. In this study, the high TEE group had higher weight, BMI, and % fat mass, but lower % muscle mass compared to the low TEE group. These findings suggest that the contribution of % fat mass to TEE may reflect the increased energy demands of maintaining a larger body mass. The contribution of fat mass to TEE in this study may reflect the combined effects of sustaining a larger body mass and overall energy demands of this body composition. These findings underscore the complex and multifaceted relationships between body composition and energy expenditure in older adults. Age-related changes in energy metabolism, such as reduced resting metabolic rates and shifts in lean tissue metabolic activity [35], may further influence these dynamics. Future research should incorporate detailed assessments of energy expenditure and metabolic costs across diverse older adult populations to better understand these intricate relationships.

In theory, one would anticipate that energy intake would increase following increased physical activity and energy expenditure [30, 69]. Therefore, current guidelines suggest that older adults should increase their physical activity to improve their appetite and energy intake [70]. While some studies have shown no significant difference between average daily energy intake and energy expenditure or physical activity [65, 71, 72], most studies suggest a positive association between energy expenditure and energy intake in young adults [73–75]. Additionally, the positive association of lean body mass with energy intake has been previously reported in young adults [9, 11, 74]. In older adults, this relationship has also been observed, with increases in fat-free mass linked to higher *ad libitum* energy intake and increased postprandial appetite [8]. A recent study in older adults further found that total daily energy intake correlates with fat-free mass and total daily energy expenditure, but not with fat mass [37]. In our study, we build on this by specifically examining the relationship between % muscle mass and energy intake in older adults. Our results suggest that higher energy expenditure and higher % muscle mass are associated with higher energy intake in older adults. Considering these findings, it may be suggested that an increase in energy expenditure and muscle mass could have a positive effect on energy intake. However, while our study provides new insights into the role of muscle mass in energy intake, it is important to note that it is cross-sectional in design. Therefore, controlled clinical trials are needed before making specific recommendations for older adults.

In addition to energy intake, some studies have also examined the relationship between physical activity levels and macronutrient intakes. A study involving younger and older individuals showed no difference in macronutrient intake based on physical activity status [65]. Similarly, a systematic review of adults also suggested no solid evidence that increased physical activity affects macronutrient intake [72]. However, another study by Camoes et al., which included subjects aged 18–92 years, found that active males had lower levels of protein intake (as a percentage of energy) but higher mean energy intakes (kcal) compared to sedentary males [76]. In our study, we observed that individuals with higher physical activity levels tended to have greater fibre intake, while those in the high total energy expenditure group had both higher energy and protein intake. This higher protein consumption in the high total energy expenditure group may reflect a higher overall energy intake or a tendency towards more nutrient-dense diet. Furthermore, given that we did not observe differences in overall energy intake between physical activity level groups, this may relate to the satiating effects of fibre, suggesting that higher physical activity levels might be associated with a more nutrient-dense diet. Future studies should investigate the specific dietary patterns associated with different levels of physical activity and energy expenditure to better understand these relationships.

It is well established that physical activity has the ability to modulate appetite regulation by enhancing the sensitivity of the physiological satiety signalling system, modifying macronutrient preferences or food choices, and changing the hedonic response to food [77]. Nonetheless, a study examining the effects of habitual physical activity on energy intake and appetite in young and older adults showed that habitual physical activity did not affect sensitivity to hunger and satiety in both group [78]. Conversely, a recent meta-analysis showed that physical activity and exercise may increase the levels of resting hunger in older adults [27]. However, it should be noted that this meta-analysis focused on limited literature due to the lack of available studies, and exercise and physical activity studies were considered equal. Therefore, it is challenging to conclusively state that high physical activity increases appetite in older adults. On the other hand, our study did not observe any significant difference in appetite between the accelerometer measured physical activity or TEE groups. We only found that females had a higher desire to eat compared to males in both groups. This might be because, while all four measures of appetite, hunger, fullness, prospective consumption, and desire to eat, are related, they reflect different aspects. The desire to eat potentially being more influenced by psychological factors, whereas the others relate more closely to physiological signals of satiety [79]. Additionally, this difference in females may stem from hormonal influences [80] or variations in emotional eating patterns [81], which can affect appetite perceptions. It is important to note that measuring appetite and energy intake objectively in free-living situations is challenging, and our study relied on self-report methods. Therefore, future intervention studies should be conducted to further explore the interplay between these factors.

To our knowledge, this is the first study to compare the association of both physical activity level and TEE with energy intake and appetite in older adults. However, there are some limitations of the current study that need to be considered. This study was conducted during the COVID-19 pandemic, which led to increased time spent at home and a rise in sedentary behaviours for many individuals [82]. Despite this, the high AMPA and TEE values observed in our cohort suggest that our participants remained quite active. This may reflect a self-selection bias, where individuals who were already more active or health-conscious were more likely to participate in the study, potentially limiting the generalisability of our findings to the broader older adult population. Additionally, data collection occurred predominantly during the winter season, which may have influenced physical activity levels, though this effect seems to have been mitigated by the particularly active nature of our sample. Another potential limitation is that while accelerometers were used to measure physical activity and assign participants to groups, these devices have limitations in capturing certain types of activities, such as resistance training, strength exercises, or cycling. We also acknowledge the limitations of self-reported dietary intake data, which is prone to underreporting or inaccuracies. This is particularly relevant in our study, where a comparison of TEE and self-reported energy intake data suggests a substantial energy deficit. Given that our participants were not of low BMI, and we excluded those with substantial weight loss in the previous 3 months, this discrepancy likely reflects the challenges associated with self-reported intake data. Although participants were provided with comprehensive written, video recording, and verbal instruction by the researchers to minimize underreporting, it remains a common issue in dietary assessments, particularly among older adults [83]. Future research should consider more objective methods for assessing energy intake to ensure greater accuracy. Additionally, in this study, some participants across different groups showed only minor differences in TEE and AMPA. Therefore, for future studies, it is important to consider that grouping participants into tertiles may reduce the likelihood of detecting differences between groups. Additionally, we acknowledge that some of the previous studies have used more robust objective measures of energy expenditure [37, 84]. However, due to challenges posed by COVID-19, we relied on self-reported methods in this study.

## Conclusion

In conclusion, our findings suggest a positive correlation between TEE and % muscle mass, as well as intakes of energy, macronutrients, and fibre. Higher TEE was associated with higher energy and protein intake, and there was a trend toward higher fibre intake in more physically active individuals. No significant differences in overall appetite measures from VAS were observed between the AMPA or TEE groups, though females reported a higher desire to eat compared to males. While percentage muscle mass was positively correlated with TEE, percentage fat mass emerged as the stronger predictor of TEE after adjusting for age and sex. These results highlight the complex relationship between body composition and energy expenditure in older adults, particularly the influence of percentage fat mass on TEE. This underlines the importance of targeting both physical activity and body composition in interventions aimed at improving energy intake in older adults.

## Data Availability

The datasets analysed during the current study are available from the corresponding author on reasonable request.

## Abbreviations

BMI: Body Mass Index
AMPA: Physical Activity Level Measured by Accelerometer
TEE: Total Energy Expenditure
sPAR: Simplified Physical Activity Record Paper
CNAQ: Council on Nutrition Appetite Questionnaire
TFEQ: Three Factor Eating Questionnaire
DEBQ: Dutch Eating Questionnaire
GPPAQ: General Practice Physical Activity Questionnaire
GNKQ: General Nutrition Knowledge Questionnaire
GFI: Groningen Frailty Indicator Questionnaire
CoEQ: Control of Eating Questionnaire
PASE: Physical Activity Scale for the Elderly
VAS: Visual Analogue Scale
MET: Metabolic Equivalent of Task
AUC: Area Under The Curve
ANCOVA: Analysis of Covariance
DEXA: Dual-energy X-ray absorptiometry
